# Fragile X syndrome: a pilot proton magnetic resonance spectroscopy study in premutation carriers

**DOI:** 10.1186/1866-1955-4-23

**Published:** 2012-08-30

**Authors:** Brian P Hallahan, Eileen M Daly, Andrew Simmons, Caroline J Moore, Kieran C Murphy, Declan D G Murphy

**Affiliations:** 1 Department of Psychiatry, National University of Ireland Galway, Galway, Ireland; 2 Section of Brain Maturation, Department of Forensic and Neurodevelopmental Science, Institute of Psychiatry, King’s College London, London, UK; 3 Department of Neuroimaging, Institute of Psychiatry, King’s College London, London, UK; 4 NIHR Biomedical Research Centre for Mental Health, London, UK; 5 Department of Psychiatry, Royal College of Surgeons in Ireland, Beaumont Hospital, Dublin, Ireland

**Keywords:** Fragile X, Premutation carriers, MRS, NAA

## Abstract

**Purpose:**

There is increasing evidence that neurodevelopmental differences in people with Fragile X syndrome (FraX) may be explained by differences in glutamatergic metabolism. Premutation carriers of FraX were originally considered to be unaffected although several recent reports demonstrate neuroanatomical, cognitive, and emotional differences from controls. However there are few studies on brain metabolism in premutation carriers of FraX.

**Methods:**

We used proton magnetic resonance spectroscopy to compare neuronal integrity of a number of brain metabolites including N-Acetyl Aspartate, Creatine + Phosphocreatinine, Choline, myoInositol, and Glutamate containing substances (Glx) in 17 male premutation carriers of FraX and 16 male healthy control individuals.

**Results:**

There was no significant between-group difference in the concentration of any measured brain metabolites. However there was a differential increase in N-acetyl aspartate with aging in premutation FraX individuals compared to controls.

**Conclusions:**

This is the first ^1^ H-MRS study to examine premutation FraX individuals. Although we demonstrated no difference in the concentration of any of the metabolites examined between the groups, this may be due to the large age ranges included in the two samples. The differential increase in NAA levels with aging may reflect an abnormal synaptic pruning process.

## Background

Expanded trinucleotide repeats are associated with several disabling neuropsychiatric and neurological conditions, such as Huntington’s disease, myotonic dystrophy, Freidreich ataxia, spinal and bulbar palsy, and Fragile X syndrome (FraX). The clinical phenotype of FraX is caused by an expansion of a single trinucleotide repeat sequence (CGG) in the 5’ untranslated region of the Fragile X Mental Retardation 1 (FMR-1) gene on the X chromosome. Expansion of the FMR1 gene to more than 200 CGG repeats (full mutation) is accompanied by methylation of FMR-1 and loss of FMR-1 protein (FMRP) production [1,2]. Premutation carriers of FraX have 55 to 200 CGG trinucleotide repeats with diminished production of FMRP in blood [3], and elevated levels of FMR-1 mRNA [4,5]. Normal controls have less than 55 CGG repeats.

The cognitive and behavioral phenotype of the full mutation of FraX has been described by many authors [6,7]. In brief, the cognitive phenotype of males usually includes a moderate to severe intellectual disability [8,9]; deficits in executive function, short-term memory, attentional control, and arithmetic and visuo-spatial processing [10]; and the behavioral phenotype includes gaze aversion, anxiety, hyperactivity, and social-interaction deficits [11]. Females with FraX are less severely affected, owing to the second, unaffected X chromosome. They may be of average intelligence or have a mild intellectual disability and often have executive function deficits [12]. They are also at an increased risk for mood disorders and social anxiety [8,13].

Contrary to initial beliefs that premutation carriers of FraX were unaffected, there is increasing evidence that they have a variety of cognitive deficits, differences in brain anatomy and that some develop Fragile X-associated tremor/ataxia syndrome (FXTAS). For example, we reported that adult male premutation carriers display a wide range of executive function, memory, attention, language and perceptual abnormalities [14], and have reduced regional grey matter volume in a number of brain regions including the cerebellum, amygdalo-hippocampal complex, caudate nucleus, and parietal lobe [15]. FXTAS is clinically characterized by cerebellar ataxia, tremor, parkinsonism, and mild cognitive decline, and occurs in 40 % to 45 % of male premutation carriers of FraX aged 50 years or older [4,16]. Age-related cognitive decline has also been documented however in male premutation carriers over 50 years of age without FXTAS, with particular age-related deficits in executive function noted [17]. Female premutation carriers of FraX similarly demonstrate abnormalities including a mild form of the physical phenotype of FraX [12,18], elevated levels of follicle stimulating hormone [19], premature ovarian insufficiency (POI) [20,21], and increased emotional problems, with high rates of major depressive disorder [13] and some anxiety disorders, in particular panic disorder and agoraphobia [22,23]. Although less common, FXTAS has also been reported in female premutation carriers of FraX [24]. Hence premutation carriers of FraX have a wide range of physical and cognitive abnormalities, many of which are associated with or become more prominent with aging.

Differences in neuronal integrity/metabolism may help explain some of these cognitive abnormalities in premutation carriers of FraX. One technique used to measure neuronal integrity is *in vivo* proton magnetic resonance spectroscopy (^1^ H-MRS) [25,26]. This provides spectra which can be used to quantify a range of brain metabolites, including N-acetyl Aspartate (NAA), Creatine + Phosphocreatine (Cr + PCr), Choline (Cho), myo-Inositol (mI), and Glutamate containing substances (Glx, which includes the combined signal from (Glutamate (Glu) and Glutamine (Gln)).

NAA is present at high concentrations in both gray and white matter. Its synthesis is closely correlated with mitochondrial energy metabolism - and so NAA is often used as a measure of neuronal density and/or mitochondrial function [26-30]. In contrast Cr + PCr and Cho are used as measures of (respectively) phosphate metabolism and membrane turnover [25,26,31-33], while mI is associated with glial cell structure and proliferation [34].

Glutamate is the major excitatory neurotransmitter and is converted into glutamine by glutamine synthetase [35]. It has been suggested that neurological and psychiatric symptoms associated with FraX may be a consequence of an exaggerated response to metabotrophic glutamate receptor (mGluR) activation due to an absence/reduction of FMRP [36]. FMRP modulates dendritic maturation and synaptic plasticity and one of the mechanisms postulated for this effect is its inhibition of the metabotrophic Glu receptors (mGluR), mGluR1 and mGluR5 mediated mRNA translation in dendrites [37,38]. In mouse models the mGluR5 antagonist 2-methyl-6-phenylethynyl-pyridine (MPEP) has been shown to reverse behavioral phenotypes (including hyperactivity, seizures, pre-pulse inhibition deficits, repetitive behaviors) and to lead to remarkable improvements in synaptic plasticity and spine morphology [39]. Furthermore, a recent human study investigated AFQ056, a receptor subtype-selective inhibitor of mGluR5, and noted an improvement in behavioral symptoms in 30 male individuals with FraX [40].

**Figure 1:**
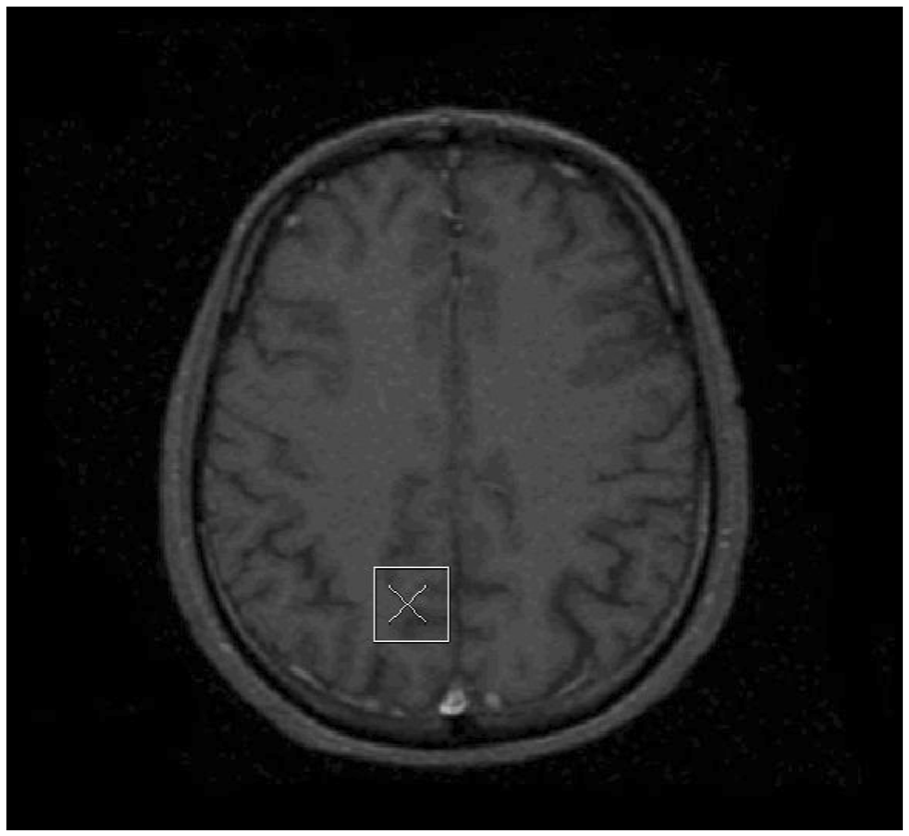
Position of voxel in the right parietal lobe.

Hence there is increasing evidence that abnormalities in glutamatergic metabolism may underpin neurodevelopmental and/or behavioral abnormalities in people with FraX. Relatively few studies, however, have investigated neuronal integrity in premutation FraX carriers using ^1^ H-MRS with only two case series (both consisted of two FXTAS individuals respectively) published to our knowledge [41,42]. Also nobody has reported on glutamate containing substances. In this study, we therefore used ^1^ H-MRS to assess neuronal integrity in the parietal lobe of premutation carriers (without FXTAS) and controls. Furthermore, we investigated age-related differences in neuronal integrity between the groups. We chose the right parietal lobe (Figure 1) as our region of interest, as we have previously demonstrated developmental differences in this brain region and it provides good signal-to-noise [15]. We chose individuals without FXTAS, as FXTAS affects only some premutation carriers over 50 years of age, is associated with significant cognitive decline and hence the inclusion of these individuals may have impacted on the validity of our results.

## Method

### Subjects

We included 17 male premutation carriers of FraX (mean age 50 ± 15, range 19–70 years) who were recruited from genetic services throughout Britain (Guy’s Hospital London; Kennedy-Galton Centre, Harrow, London; St. James Hospital, Leeds; Wessex Hospital, Southampton) on the basis of their genotype (that is, not phenotype). The mean, SD, and age range (47 ± 17, range, 20–70 years) of the 16 healthy control subjects did not differ from the premutation carriers. Similarly they did not differ in IQ or in handedness and were recruited locally through advertisement and underwent the same investigations as the premutation carriers of FraX. All participants gave informed consent and/or assent (as approved by the Institute of Psychiatry and the South London and Maudsley NHS Trust research ethics committee and the individual local research ethics committees attached to the genetic centers where subjects were recruited).

All participants in the study underwent routine blood tests and a structured physical and psychiatric examination [43,44], (for the presence of DSM-IV axis 1 or 2 disorder: [45]). Full scale intelligence quotient (IQ) was measured by the Wechsler Adult Intelligence Scale [46], and handedness was determined using Annett’s questionnaire [47]. We used a variety of psychometric tests to further assess psychopathology including depression - Beck Depression Inventory (BDI-II) [48], anxiety - Beck Anxiety Inventory (BAI) [49] and the Hamilton Anxiety Scale (HAS) [50], and obsessionality - Yale-Brown obsessive compulsive scale (Y-BOCS) [51].

Individuals were excluded if they had a history of major psychiatric disorder (for example, psychosis), autism spectrum disorder, head injury, epilepsy, toxic exposure, abnormalities in routine blood tests, drug or alcohol misuse, clinical abnormality on routine MRI, or other genetic disorders. All subjects were familiarized with the MRI scanner before imaging and no sedation was used during the scanning process.

### Blood/cheek swab testing

Polymerase chain reaction (PCR) analysis [52], confirmed premutation FraX carrier (55–200 CGG trinucleotide repeats), and control (<55 CGG trinucleotide repeats) status and a ‘Fragile X Size Polymorphism Assay’ kit (Applied Systems) measured the precise CGG trinucleotide repeat number.

### MRI and MRS acquisition

All 17 male premutation FraX subjects, and 16 male controls underwent MRI scanning on the same 1.5-T GE Signa NV/i MR system (General Electric, Milwaukee, WI, USA) at the Maudsley Hospital, London. A 3D fast inversion-recovery prepared spoiled gradient acquisition in the steady state (IR-FSPGR) pulse sequence was acquired from all subjects with inversion time = 450 ms, echo time = 2.8 ms, and repetition time = 13.8 ms using acquisition parameters chosen using a contrast simulation tool [53]. There were 124 contiguous coronal slices acquired with a slice thickness of 1.5 mm and a matrix of 256x256, resulting in an in-plane resolution of 0.859x0.859 mm^2^.

**Figure 2:**
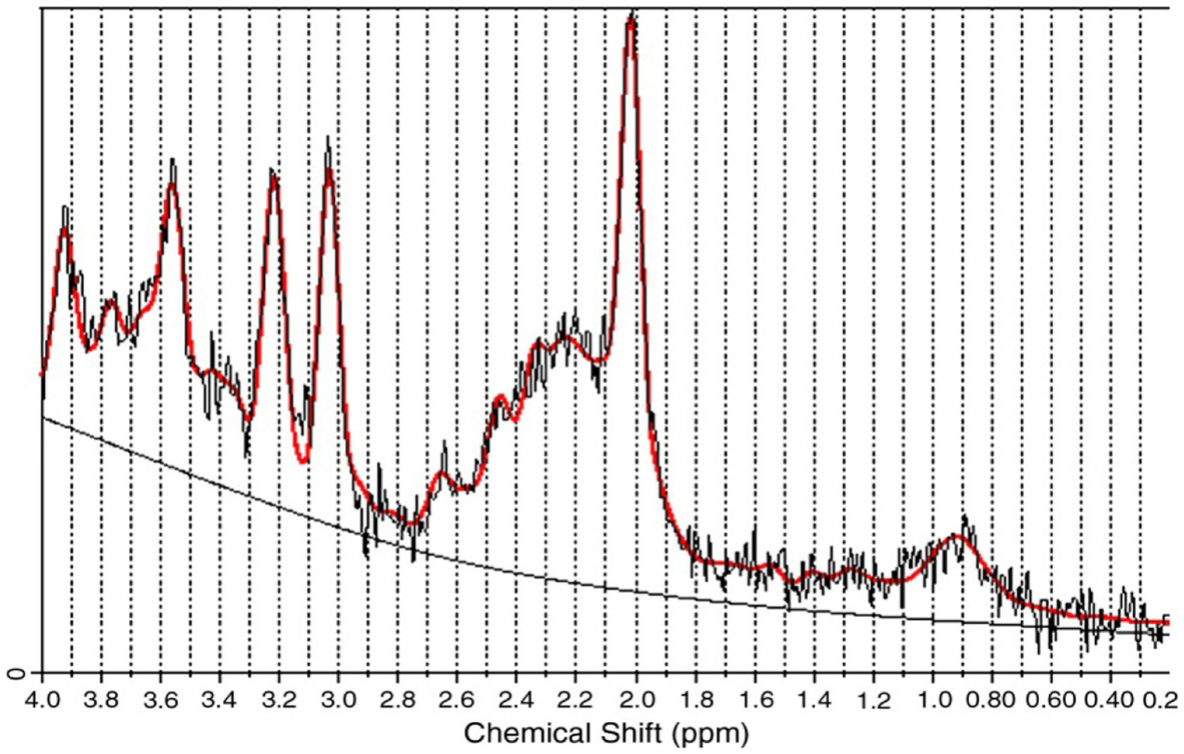
**An example of typical MR spectra from the right parietal lobe.** The *in vivo* data and fitted baseline are shown in black, with the LCModel fit overlaid in red.

Single-voxel ^1^ H MR spectroscopy was performed in the same scanning session using a point-resolved spectroscopy (PRESS) sequence with repetition time = 3 s, echo time = 35 ms, and 160 averages with automated shimming and water suppression and excellent reproducibility [54] and used to obtain spectra from each voxel after CHESS water suppression with high signal to noise ratio and clearly resolved NAA, Cho, mI, and Cr + PCr peaks among other metabolites. MR spectra were acquired from a 6 mL volume prescribed over the right parietal lobe using co-ordinates derived from the coronal IR-FSPGR images (Figure 1). The water suppression and shimming were optimized using a standard automated pre-scan, and *in-vivo* metabolite levels for NAA, Cr + PCr, Glx, Cho, and mI were measured using LCModel software (LCModel version 6.1) [55]. Each spectra was reviewed to ensure adequate signal to noise ratio (≥6 as determined by LCModel) and line width (maximum 10 Hz), as well as the absence of artifacts. The Cramer Rao lower bound was chosen to be < 20 to ensure a good quality fit for each peak. LCModel uses a linear combination of model spectra of metabolite solutions in vitro to analyze the major resonances of *in vivo* spectra. In this case, a basis set acquired on the scanner consisting of alanine, aspartate, creatine, gamma-aminobutyric acid (GABA), glutamine, glutamate, glycerophosphocholine, mI, lactate, NAA, N-acetyl-aspartylglutamate (NAAg), scyllo-inositol, and taurine, was used, together with a baseline function. Lipids and macromolecules were not estimated. An example LCModel output is given in Figure 2

To ensure that differences in tissue composition did not account for metabolic differences between subject groups the tissue composition of each ^1^ H-MRS voxel was analyzed using SPM (Statistical Parametric Mapping) software (available at http://www.fil.ion.bpmf.ac.uk/spm) to determine the percentage of grey and white matter and CSF composition from the IR-FSPGR images within the MRS voxel. The metabolite levels were then corrected for the amount of cerebrospinal fluid in the spectroscopy voxel.

As expected, many of the metabolite peaks that were included in the LC model had a Cramer-Rao lower bound of > 20. However, NAA, Cr + PCr, Cho, mI, and Glx all had a Cramer-Rao lower bound < 20 for the parietal voxel of interest, and concentrations were therefore derived from these metabolite peaks.

All spectroscopy analysis was carried out blind to subject status.

### Statistical analysis

#### Spectroscopy

All spectroscopy data were normally distributed. We compared mean differences in metabolite concentrations between premutation carriers of FraX and controls using independent t-tests. We also correlated (within each group) age and metabolite concentrations. We then investigated group differences in brain aging by transforming the relevant Pearson’s *r* coefficient into Fisher’s *Z*-score to test the significance of the difference between correlations, where a *Z*_observed_ ≥ ±1.96 is significant [56].

## Results

### Demographic profile

There was no between group difference in age or IQ at the time of MRI acquisition between premutation FraX carriers and controls. Similarly no difference was noted between the groups in symptoms of depression, anxiety, or obsessive compulsive disorder (Table 1).

**Table 1:** Psychometric data

	**Premutation FraX Carriers (*n*** **= 17) Mean (SD)**	**Healthy controls (*n*** **= 16) Mean (SD)**	***P***
Age (years)	50 (15)	47 (17)	0.547
Full Scale IQ	114 (13)	115 (17)	0.882
Verbal IQ	113 (16)	116 (21)	0.669
*Subcategories*	51 (9)	53 (14)	0.570
Vocabulary	26 (4)	26 (5)	0.947
Comprehension	21 (3)	21 (5)	0.832
Similarities	113 (12)	110 (14)	0.552
*Performance IQ*			
Block design	35.50 (8.00)	32.82 (8.34)	0.413
Object Assembly	28.81 (6.02)	25.91 (6.39)	0.249
BDI	5 (5)	5 (4)	0.746
BAI	5 (6)	3 (2)	0.276
HAS	0 (0)	0 (0)	1.000
Y-BOCS - rituals	2 (4)	2 (4)	0.910
Y-BOCS - obsessions	3 (4)	3 (4)	0.910
CGG	87 (18)	29 (6)	< 0.00001

BAI,Beck Anxiety Inventory, BDI, Beck Depression Inventory, CGG, Cytosine-guanine-guanine, HAS, Hamilton Anxiety Scale, Y-BOCS, Yale Brown Obsessive Compulsive Scale.

### Spectroscopy (Tables 2 and 3)

**Table 2:** Spectroscopy data

	**Premutation FraX Carriers (*n*** **= 17) Mean (SD)**	**Healthy controls (*n*** **= 16) Mean (SD)**	***P***
Voxel contents			
Grey matter volume %	42.4 (12.8)	40.5 (7.8)	0.605
White matter volume %	52.1 (14.1)	54.1 (8.8)	0.627
CSF matter volume %	5.5 (3.0)	5.4 (1.9)	0.928
Choline (mM)	1.23 (0.16)	1.20 (0.20)	0.598
Creatine + phosphocreatinine (mM)	6.17 (0.39)	5.90 (0.41)	0.062
Myoinositol (mM)	3.90 (0.40)	3.83 (0.53)	0.665
NAA (mM)	6.21 (0.42)	6.09 (0.81)	0.600
Glx (mM)	9.95 (0.17)	9.91 (0.28)	0.430

All spectroscopy data attained from voxel in right parietal lobe.

Glx, Glutamate/Glutamine; NAA, N-acetyl aspartate.

**Table 3:** Correlation of brain metabolites and age in premutation FraX carriers and controls

	**Correlation with age**		**Difference between the two group z**
	**Premutation FraX Carriers (*n*** **= 17) r**	**Healthy controls (*n*** **= 16) r**	
Choline	0.002	−0.210	0.548
Creatine + phosphocreatinine	0.274	0.077	0.530
Myoinositol	0.375	0.243	0.379
NAA	0.303	−0.412	2.345*
Glx	−0.024	−0.585	1.678

The difference in correlation co-efficients between premutation FraX carriers and controls is expressed where *Z* ≥ ±1.96 is significant (*P* < 0.05).

There was no difference in the content of grey matter, white matter or cerebrospinal fluid (CSF) in the voxel of interest between premutation FraX individuals and controls. Similarly no difference was noted in the concentration of any of the metabolites measured.

**Figure 3:**
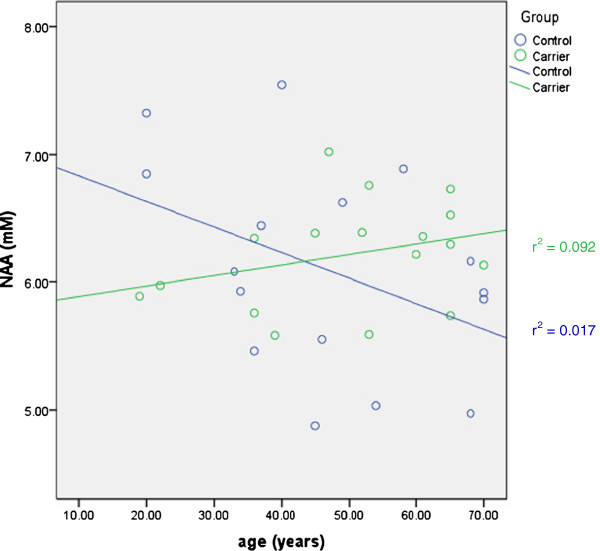
Correlation between NAA levels measured in the right parietal lobe and age in premutation FraX carriers and Controls.

**Figure 4:**
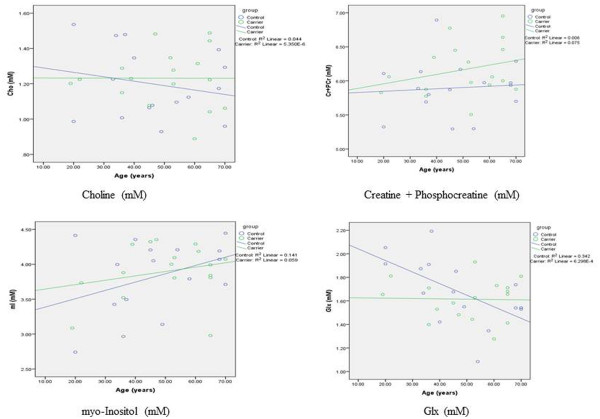
Correlation between Cho, Cr + PCr, mI and Glx measured in the right parietal and age in premutation Frax Carriers and Controls.

A differential increase in NAA levels with aging within premutation FraX carriers was found compared to controls (Z = 2.345) (Figure 3). A non-significant differential increase in Glx levels with aging within premutation carriers compared to controls was noted (z = 1.678) (Figure 4). When we examined individuals between 30 and 60 years of age only (*n* = 18), this differential increase in Glx levels was significant (z = 2.110).

## Discussion

This is the first MRI spectroscopy study to compare a group of pre-mutation FraX individuals to healthy controls. We demonstrated an age-related increase in NAA in the pre-mutation FraX individuals compared to healthy controls. We found no mean difference in the concentration of any of the metabolites examined between the two groups, although this may be due to the large age ranges included in the two samples.

Two previous studies (examining four individuals in total) with FXTAS gave conflicting results in relation to NAA levels with two individuals having reduced cerebellar NAA/Cr + PCr levels and two individuals having normal NAA/Cr + PCr levels [41,42]. There have been no previous MR spectroscopy studies to our knowledge to date examining full mutation FraX individuals.

NAA is present at high concentrations in both grey and white matter and its’ synthesis is closely correlated with mitochondrial energy metabolism - therefore NAA is often used as a measure of neuronal density and/or mitochondrial function [26-30]. NAA levels in healthy controls usually increase gradually with age in grey and white matter until approximately the first and third decades respectively before declining gradually thereafter [57,58]. Thus, increased NAA levels, with subsequent down-regulation, are normally associated with healthy cerebral maturation [59]. Morphological studies of healthy neural development are generally consistent with these spectroscopic findings, reporting an initial rapid overproduction of neural synapses in early to late childhood [60], with subsequent synapse elimination late in childhood and adolescence [61], followed by a slow reduction in synaptic density thereafter [61,62]. Our finding of a differential increase in NAA concentration with aging in premutation carriers of FraX is consistent with previous research suggesting an abnormal maturation process; for example increased cell packing secondary to greater synaptic density. This suggestion is supported by prior work in mice [63], Drosophilia [64], and humans [65,66], that demonstrated synaptic alterations in the absence of FMRP. Alternatively, the greater age-related increase in NAA in premutation carriers of FraX may reflect a hyper-metabolic state in the mitochondria with a subsequent increased risk of oxidative damage and neuronal apoptosis [67].

We detected no difference in Glx levels and found no age-related changes in these metabolites between the FraX and control groups, however we were unable to examine these metabolites (that is, Glu/Gln) individually as these can only be reliably examined at magnetic field strengths > 3 T due to the multiple overlap of the resonances at field strengths < 3 T. Therefore future studies, at higher magnetic field strengths that can examine these metabolites individually are merited. Indeed, future pharmaco-therapeutic strategies for FraX may focus on Glu, with evidence that the mGluR5 antagonist, MPEP, abolishes the audiogenic seizure phenotype in *FMR1* knock-out mice [68], decreases the mushroom body defects (fused β-lobes) [69], and as described above, ameliorates several clinical features in mouse models. These findings have been replicated in multiple animal models with many phenotypes and have led to several human Phase II trials that are ongoing.

In addition to a medium field strength (1.5 T), our study was also relatively small and so may not have had sufficient power to detect small group differences. However, it is the largest study to date in this population, and we have reported case–control differences in similar sample sizes in people with other neurodevelopmental disorders. We did not obtain spectra from other brain regions (due to time constraints) and thus we were unable to examine the neuronal integrity of limbic regions and cerebellum for example. Also, this is a cross-sectional study, and therefore we are only able to describe age-related differences - and not individual changes over time. Hence, there may have been undetectable age-related confounders (for example, health differences) affecting our results, although all individuals had no recent health difficulties at the time of scanning. Nevertheless, this pragmatic study design allows analysis across a wide age range (52 years), which would not be achievable in a longitudinal brain imaging study. Whilst we found no difference in NAA levels between the two groups, NAA levels varied with age in both groups and therefore it is possible that if we examined a narrower age range and had greater numbers of individuals in the study, differences in mean NAA levels may have been detected between premutation FraX individuals and controls. Consequently our finding of no difference in the levels of NAA should be interpreted with caution. Similarly, given the large age range and relatively small sample size, our findings of no difference in the levels of other metabolites should be interpreted with caution.

## Conclusion

This is the first ^1^ H-MRS study to examine premutation FraX individuals. We detected a differential increase in NAA levels with aging. This finding provides initial evidence for an abnormal maturation process in permutation FraX - perhaps reflecting increased cell packing secondary to abnormalities in synaptic pruning and synaptic density; and/or differences in mitochondrial metabolism.

## Competing interests

The authors declare that they have no competing interests.

## Authors’ contributions

BH, ED, CM, and AS were involved in the data acquisition and image analysis. BH, ED, KM, MC, AS, and DM were involved in the study conception and design. BH, MC, ED, AS, KM, and DM, were involved in data interpretation. All authors were involved in approval of the article to be published.

## References

[B1] Verkerk AJMH Pieretti M Sutcliffe JS Fu Y-H Kuhl DPA Pizzuti A Reiner O Richards S Victoria MF Zhang F Eussen BE van Ommen GJB Blonden LAJ Riggins GJ Chastain JL Kunst CB Galjaard H Caskey CT Nelson DL Oostra BA Warren ST Identification of a gene (FMR-1) containing a CGG repeat coincident with a breakpoint cluster region exhibiting length variation in Fragile-X syndrome Cell 1991 65 905 914 10.1016/0092-8674(91)90397-H 1710175

[B2] Yu S Pritchard M Kremer E Lynch M Nancarrow J Baker E Holman K Mulley JC Warren ST Schlessinger D Fragile X genotype characterized by an unstable region of DNA Science 1991 252 1179 1181 10.1126/science.252.5009.1179 2031189

[B3] Tassone F Haegerman RJ Taylor AK Mills JB Harris SW Gane LW Hagerman PJ Clinical involvement and protein expression in individuals with the FMR1 premutation Am J Med Genet 2000 91 144 152 10.1002/(SICI)1096-8628(20000313)91:2<144::AID-AJMG14>3.0.CO;2-V 10748416

[B4] Hagerman RJ Leehey M Heinrichs W Tassone F Wildon R Hills J Grigsby J Gage B Hagerman PJ Intention tremor, parkinsonism, and generalised brain atrophy in male carriers of fragile X Neurology 2001 57 127 130 10.1212/WNL.57.1.127 11445641

[B5] Tassone F Haegerman RJ Taylor AK Gane LW Godfrey TE Haegerman PJ Elevated levels of FMR1 mRNA in carrier males; a new mechanism of involvement in the fragile-X syndrome Am J Hum Genet 2000 66 6 15 10.1086/302720 10631132 PMC1288349

[B6] Bennetto L Pennington BF Hagerman RJ, Hagerman PJ Neuropsychology Fragile X syndrome: Diagnosis, treatment and research 2002 3 The Johns Hopkins University Press, Baltimore

[B7] Turk J The fragile X syndrome: on the way to a behavioural phenotype Br J Psychiatry 1992 160 24 35 10.1192/bjp.160.1.24 1544010

[B8] Gothelf D Furfaro JA Penniman LC Glover GH Reiss AL The contribution of novel brain imaging techniques to understanding the neurobiology of mental retardation and developmental disabilities MRDD Research Reviews 2005 11 331 339 10.1002/mrdd.20089 16240408

[B9] Hessl D Nguyen DV Green C Chavez A Tassone F Hagerman RJ Senturk D Schneider A Lightbody A Reiss AL Hall S A solution to limitations of cognitive testing in children with intellectual disabilities: the case of fragile X syndrome J Neurodevelop Disord 2008 1 33 45 10.1007/s11689-008-9001-8 PMC2768415 19865612

[B10] Schapiro MB Murphy DG Hagerman RJ Azari NP Alexander GE Miezejeski CM Hinton VJ Horwitz B Haxby JV Kumar A White B Grady CL Adult fragile X syndrome: neuropsychology, brain anatomy, and metabolism Am J Med Genet 1995 60 480 493 10.1002/ajmg.1320600603 8825884

[B11] Levitas A Neuropsychiatric aspects of fragile X syndrome Semin Clin Neuropsychiatry 1996 1 154 167 10320415 10.1053/SCNP00100154

[B12] Riddle JE Cheema A Sobesky WE Gardner SC Taylor AK Pennington BF Hagerman RJ Phenotypic involvement in females with the FMR1 gene mutation Am J Ment Retard 1998 102 590 601 9606468 10.1352/0895-8017(1998)102<0590:piifwt>2.0.co;2

[B13] Roberts JE Bailey DB Mankowski J Ford A Sideris J Weisenfeld LA Heath TM Golden RN Mood and anxiety disorders in females with the FMR1 premutation Am J Med Genet 2009 150B 130 139 10.1002/ajmg.b.30786 18553360

[B14] Moore CJ Daly E Schmitz N Tassone F Tysoe C Hagerman RJ Hagerman PJ Morris RG Murphy KC Murphy DG A neuropsychological investigation of male premutation carriers of fragile X syndrome Neuropsychologia 2004 42 1934 1947 10.1016/j.neuropsychologia.2004.05.002 15381024

[B15] Moore CJ Daly EM Tassone F Tysoe C Schmitz N Ng V Chitnis X McGuire P Suckling J Davies KE Hagerman RJ Hagerman PJ Murphy KC Murphy DGM The effect of pre-mutation of X chromosome CGG trinucleotide repeats on brain anatomy Brain 2004 127 2672 2681 10.1093/brain/awh256 15483045

[B16] Jacquemont S Hagerman RJ Leehey M Grigsby J Zhang L Brunberg JA Greco C Des Portes V Jardini T Levine R Berry-Kravis E Brown WT Schaeffer S Kissel J Tassone F Hagerman PJ Fragile X premutation tremor/ataxia syndrome: molecular, clinical and neuroimaging correlates Am J Hum Genet 2003 72 869 878 10.1086/374321 12638084 PMC1180350

[B17] Cornish KM Kogan CS Li L Turk J Jacquemont S Hagerman RJ Lifespan changes in working memory in fragile X premutation males Brain Cogn 2009 69 551 558 10.1016/j.bandc.2008.11.006 19114290 PMC4158922

[B18] Hull C Hagerman RJ A study of the physical, behavioral, and medical phenotype, including anthropometric measures of females with fragile X syndrome Am J Dis Child 1993 147 1236 1241 8237919 10.1001/archpedi.1993.02160350110017

[B19] Hundscheid RD Braat DD Kiemeney LA Smits A Thomas CM Increased serum FSH in female fragile X premutation carriers with either regular menstrual cycles or on oral contraceptives Hum Reprod 2001 16 457 462 10.1093/humrep/16.3.457 11228211

[B20] Allingham-Hawkins DJ Babul-Hirji R Chitayat D Holden JJ Yang KT Lee C Hudson R Gorwill H Nolin SL Glicksman A Jenkins EC Brown WT Howard-Peebles PN Becchi C Cummings E Fallon L Seitz S Black SH Vianna-Morgante AM Costa SS Otto PA Mingroni-Netto RC Murray A Webb J Vieri F Krekewich K Humphries T Fragile X premutation is a significant risk factor for premature ovarian failure. The international collaborative POF in fragile X study--preliminary data Am J Med Genet 1999 83 322 325 10.1002/(SICI)1096-8628(19990402)83:4<322::AID-AJMG17>3.0.CO;2-B 10208170 PMC3728646

[B21] Conway GS Hettitarachein S Murray A Jacobs PA Fragile X premutations in familial premature ovarian failure Lancet 1995 346 309 310 10.1016/S0140-6736(95)92194-X 7630263

[B22] Adams PE Adams JS Nguyen DV Hessl D Brunberg JA Tassone F Zhang W Koldewyn K Rivera SM Grigsby J Zhang L Decarli C Hagerman PJ Hagerman RJ Psychological symptoms correlate with reduced hippocampal volume in fragile X premutation carriers Am J Med Genet B Neuropsychiatr Genet 2010 153B 775 785 19908235 10.1002/ajmg.b.31046 PMC2868927

[B23] Cordeiro L Ballinger E Hagerman R Hessl D Clinical assessment of DSM-IV anxiety disorders in fragile X syndrome: prevalence and characterization J Neurodevelop Disord 2011 3 1 11 10.1007/s11689-011-9075-6 PMC3057014 21475730

[B24] Coffey SM Cook K Tartaglia N Tassone F Nguyen DV Pan R Bronsky HE Yuhas J Borodyanskaya M Grigsby J Doerflinger M Hagerman PJ Hagerman RJ Expanded clinical phenotype of women with the FMR1 premutation Am J Med Genet 2008 146A 1009 1016 10.1002/ajmg.a.32060 18348275 PMC2888464

[B25] Danielson ER Ross B Magnetic Resonance Spectroscopy Diagnosis of Neurological Diseases 1999 Marcel Dekker, Inc., New York, NY

[B26] Koller KJ Zacsek R Coyle JT N-acetyl-aspartyl-glutamate: regional levels in rat brain and the effects of brain lesions as determined by a new HPLC method J Neurochem 1984 43 1136 1142 10.1111/j.1471-4159.1984.tb12854.x 6470709

[B27] Bhakoo K Craig T Styles P Cellular and developmental tissue distribution of NAA catabolic enzyme, aspartoacylase. Insights into NAA Function Proc Intl Soc Mag Reson Med 2001 9 490

[B28] Brandao L Domingues RC MR Spectroscopy of the Brain 2004 Lippincott Williams & Wilkins, Philadelphia, PA

[B29] Clark JB N-acetyl aspartate: a marker for neuronal loss or mitochondrial dysfunction Dev Neurosci 1998 20 271 276 10.1159/000017321 9778562

[B30] Gill SS Thomas GT Van Bruggen N Gadian DG Peden CJ Bell JD Cox IJ Menon DK Iles RA Bryant DJ Proton NR spectroscopy of intracranial tumours: in vivo and in vitro studies J Comput Assist Tomogr 1990 14 497 504 10.1097/00004728-199007000-00001 2164536

[B31] Bruhn H Frahm J Gyngell ML Merboldt KD Hanicke W Sauter R Cerebral metabolism in man after acute stroke: new observations using localized proton NMR spectroscopy Magn Reson Med 1989 9 126 131 10.1002/mrm.1910090115 2540394

[B32] Davie CA Hawkins CP Barker GJ Brennan A Tofts PS Miller DH McDonald WI Serial proton magnetic resonance spectroscopy in acute multiple sclerosis lesions Brain 1994 117 49 58 10.1093/brain/117.1.49 8149214

[B33] Nishizuka Y The molecular heterogeneity of protein kinase C and its implications for cellular regeneration Nature 1998 334 661 665 3045562 10.1038/334661a0

[B34] Huang W Alexander G Chang L Shetty H Krasuski J Rapoport S Schapiro M Brain metabolite concentration and dementia severity in Alzheimer’s disease: a (1)H MRS study Neurology 2001 57 626 632 10.1212/WNL.57.4.626 11524470

[B35] Martinez-Hernandez A Bell KP Norenberg MD Glutamine synthetase: glial localization in brain Science 1977 195 1356 1358 10.1126/science.14400 14400

[B36] Bear MF Dolen G Osterweil E Nagarajan N Fragile X: translation in action Neuropsychopharmacology 2008 33 84 87 10.1038/sj.npp.1301610 17940551 PMC4327813

[B37] Aschrafi A Cunningham BA Edelman GM Vanderklish PW The Fragile X mental retardation protein and group I metabotropic glutamate receptors regulate levels of mRNA granules in brain Proc Natl Acad Sci USA 2005 102 2180 2185 10.1073/pnas.0409803102 15684045 PMC548595

[B38] Grossman AW Aldridge GM Weiler IJ Greenough WT Local protein synthesis and spine morphogenesis: Fragile X syndrome and beyond J Neurosci 2006 26 7151 7155 10.1523/JNEUROSCI.1790-06.2006 16822971 PMC6673953

[B39] Silverman JL Tolu S Barkan C Crawley J Repetitive self-grooming behavior in the BTBR mouse model of autism is blocked by the mGluR5 antagonist MPEP Neuropsychopharmacology 2010 35 976 989 10.1038/npp.2009.201 20032969 PMC2827881

[B40] Jacquemont S Curie A Portes V Torriolo MG Berry-Kravis E Hagerman RJ Ramos FJ Cornish K He Y Paulding C Neri G Chen F Hadjikhani N Martinet D Meyer J Beckerman JS Delange K Brun A Bussy G Gasparini F Hilse T Floesser A Branson J Bilbe G Johns D Gomez-Mancilla B Epigenetic modification of the FMR1 gene in Fragile X syndrome is associated with differential response to the mGluR5 antagonist AFQ056 Sci Transl Med 2011 3 64ra1 10.1126/scitranslmed.3001708 21209411

[B41] Ginestroni A Guerrini L Della Nave R Tessa C Cellini E Dotti MT Brunori P De Stefano N Piacentini S Mascalchi M Morphometry and 1 H-MR spectroscopy of the brain stem and cerebellum in three patients with fragile X-associated tremor/ataxia syndrome Am J Neuroradiol 2007 28 486 488 17353317 PMC7977833

[B42] Rizzo G Pizza F Scaglione C A case of Fragile X premutation tremor/ataxia syndrome with evidence of mitochondrial dysfunction Mov Disord 2006 21 1541 1542 10.1002/mds.21037 16830323

[B43] Murphy DGM Mentis MJ Pietrini P Grady G Daly E Haxby JV De La Granja M Allen G Largay K White BJ Powell CM Horwitz B Rapoport SI Schapiro MB A PET study if Turner’s syndrome: effects of sex steroids and the X chromosome on brain Biol Psychiatry 1997 41 285 298 10.1016/S0006-3223(95)00660-5 9024951

[B44] Murphy DGM Mentis MJ Pietrini P Grady CL Moore CJ Horowitz B Hinton V Dobkin CS Schapiro MB Rapoport SI Premutation female carriers of fragile x syndrome: a pilot study on brain anatomy and metabolism J Am Acad Child Adolesc Psychiatry 1999 38 1294 1301 10.1097/00004583-199910000-00019 10517063

[B45] American Psychiatric Association Diagnostic and Statistical Manual of Mental Disorders. DSM-IV 1994 4 American Psychiatric Association, Washington, DC

[B46] Wechsler D Wechsler Adult Intelligence Scale-Revised 1987 Psychological Corporation, New York, NY

[B47] Annet N A classification of hand preferences by association analysis Br J Psychol 1970 61 303 321 10.1111/j.2044-8295.1970.tb01248.x 5457503

[B48] Beck AT Beck Depression Inventory 1987 The Psychological Corporation, San Antonio, TX

[B49] Beck AT Beck Anxiety Inventory 1987 The Psychological Corporation, San Antonio, TX

[B50] Hamilton M The assessment of anxiety states by rating Br J Med Psychol 1959 32 50 55 10.1111/j.2044-8341.1959.tb00467.x 13638508

[B51] Goodman WK Price LH Rasmussen SA Mazure C Fleischmann RL Hill CL Heninger GR Charney DS The Yale-Brown Obsessive Compulsive Scale. I. Development, use and reliability Arch Gen Psychiatry 1989 46 1006 1011 10.1001/archpsyc.1989.01810110048007 2684084

[B52] Brown WT Houck GE Jeziorowska A Levinson FN Ding X Dobkin C Zhong N Henderson J Brooks SS Jenkins EC Rapid fragile X carrier screening and prenatal diagnosis using a non-radioactive PCR test JAMA 1993 270 1569 1575 10.1001/jama.1993.03510130075034 8371467

[B53] Simmons A Arridge SR Barker GJ Williams SCR Simulation of MRI cluster plots and application to neurological segmentation Magnetic Resonance Imaging 1996 14 73 92 10.1016/0730-725X(95)02040-Z 8656992

[B54] Simmons A Smail M Moore E Williams SCR Serial Precision of metabolite peak area ratios and water referenced metabolite peak areas in Proton MR spectroscopy of the human brain Magnetic Resonance Imaging 1998 16 319 330 10.1016/S0730-725X(97)00280-4 9621973

[B55] Provencher SW Estimation of metabolite concentrations from localized in vivo proton NMR spectra Magn Reson Med 1993 30 672 679 10.1002/mrm.1910300604 8139448

[B56] Pallant J SPSS survival manual. A step by step guide on data analysis using SPSS for Windows Version 11 2001 Open University Press, Maidenhead: Buckingham

[B57] Horska A Kaufmann WE Brant LJ Naidu S Jarris JC Barker PB In vivo qualitative proton MRS study of brain development from childhood to adolescence J Magn Reson Imaging 2002 15 137 143 10.1002/jmri.10057 11836768

[B58] Kadota T Horinouchi T Kuroda C Development and aging of the cerebrum: assessment with proton MR spectroscopy Am J Neuroradiol 2001 22 128 135 11158898 PMC7975536

[B59] Kreis R Ernst T Ross BD Development of the human brain: in vivo quantification of metabolite and water content with proton magnetic resonance spectroscopy Magn Reson Med 1993 30 424 437 10.1002/mrm.1910300405 8255190

[B60] Kostovic I Judas M Petanjek Z Simic G Ontogenesis of goal directed behaviour: anatomo-functional considerations Int J Psychophysiol 1995 19 85 102 10.1016/0167-8760(94)00081-O 7622411

[B61] Huttenlocher PR Synaptic density in human frontal cortex: developmental changes and effects of aging Brain Res 1979 163 195 205 10.1016/0006-8993(79)90349-4 427544

[B62] Huttenlocher PR Dabholkar AS Regional differences in synaptogenesis in human cerebral cortex J Comp Neuro 1997 387 167 178 10.1002/(SICI)1096-9861(19971020)387:2<167::AID-CNE1>3.0.CO;2-Z 9336221

[B63] Irwin SA Galvez R Greenough WT Dendritic spine structural anomalies in fragile-X mental retardation syndrome Cereb Cortex 2000 10 1038 1044 10.1093/cercor/10.10.1038 11007554

[B64] Gao FB Understanding fragile X: insights from retarded flies Neuron 2002 34 859 862 10.1016/S0896-6273(02)00740-7 12086633

[B65] Hinton VJ Brown WT Wisniewski K Rudelli RD Analysis of neocortex in three males with the fragile X syndrome Am J Med Genet 1991 41 289 294 10.1002/ajmg.1320410306 1724112

[B66] Irwin SA Idupulapati M Gilbert ME Harris JB Chakravarti AB Rogers EJ Crisostomo RA Larsen BP Mehta A Alcantara CJ Patel B Swain RA Weiler IJ Oostra BA Greenough WT Dendritic spine and dendritic field characteristics of layer V pyramidal neurons in the visual cortex of fragile-X knockout mice Am J Med Genet 2002 111 140 146 10.1002/ajmg.10500 12210340

[B67] Palmfeldt J Vang S Stengroen V Pedersen CB Christensen JH Bross P Gregersen N Mitochondrial proteomics on human fibroblasts for identification of metabolic imbalance and cellular stress Proteome Sci 2009 7 20 10.1186/1477-5956-7-20 19476632 PMC2695441

[B68] Yan QJ Rammal M Tranfaglia M Bauchwitz RP Suppression of two major Fragile X syndrome mouse model phenotypes by the mGluR5 antagonist MPEP Neuropharmacology 2005 49 1053 1066 10.1016/j.neuropharm.2005.06.004 16054174

[B69] McBride SM Choi CH Wang Y Liebelt D Braunstein E Ferreiro D Sehgal A Siwicki KK Dockendorff TC Nguyen HT McDonald TV Jongens TA Pharmacological rescue of synaptic plasticity, courtship behaviour, and mushroom body defects in a Drosophila model of fragile X syndrome Neuron 2005 45 753 764 10.1016/j.neuron.2005.01.038 15748850

